# Polymorphisms of Genes Encoding Multidrug Resistance Proteins as a Predictive Factor for Second-Line Docetaxel Therapy in Advanced Non-small Cell Lung Cancer

**DOI:** 10.1007/s12253-016-0156-4

**Published:** 2016-12-17

**Authors:** Michał Szczyrek, Radosław Mlak, Paweł Krawczyk, Kamila Wojas-Krawczyk, Tomasz Powrózek, Aneta Szudy-Szczyrek, Agnieszka Zwolak, Jadwiga Daniluk, Janusz Milanowski

**Affiliations:** 1 Department of Pneumonology, Oncology and Allergology, Medical University of Lublin, Lublin, Poland; 2 Chair of Internal Medicine and Department of Internal Medicine in Nursing, Medical University of Lublin, Lublin, Poland; 3 Department of Human Physiology, Medical University of Lublin, Lublin, Poland; 4 Chair and Department of Haematooncology and Bone Marrow Transplantation, Medical University of Lublin, Lublin, Poland

**Keywords:** NSCLC, Docetaxel, *TUBB3*, *ABCC2*, *MDR1*

## Abstract

Multidrug resistance (MDR) remains a substantial problem in chemotherapy. The purpose of the study was to investigate potential factors, including *MDR* genes polymorphisms, that could be used in qualification for second-line docetaxel therapy in non-small cell lung cancer (NSCLC) patients after failure of platinum based chemotherapy. Study group comprised of 58 Caucasian subjects. Evaluation of Single Nucleotide Polymorphisms (SNPs) of *ABCC2/MRP2* and *ABCB1/MDR1* genes was performed using the High Resolution Melting (HRM) technique. *TUBB3* gene expression was evaluated on RNA isolated from tumor tissue. Results with *p* value of <0.05 were considered significant. Factors associated with reduced risk of disease progression included good performance status (PS), long period between diagnosis and docetaxel treatment, and smoking for <10 pack-years. Disease control occurred more often in patients with G/G genotype of the *ABCC2/MRP2* gene. Median overall survival was 4.25 months. Factors such as: good PS, disease control after docetaxel, long period from diagnosis to docetaxel, lack of significant weight loss, and third-line treatment were associated with prolongation of patients survival. Overall survival probability was significantly lower in patients with significant weight loss, poor PS, lack of disease control after docetaxel, and without third-line treatment. Factors that characterized the highest risk of survival shortening were: inability to apply third-line treatment, lack of best response to first-line therapy, poor PS, and C/G or G/G genotypes of *ABCC2/MRP2* gene. We concluded that assessed factors had mainly prognostic and not predictive value. Finding reliable molecular predictors for second line docetaxel therapy requires further clinical trials.

## Introduction

Non-small cell lung cancer (NSCLC) accounts for 80% of lung neoplasms. Early stage of the disease is an indication for surgery, while locally advanced or metastatic NSCLC is treated with chemotherapy, radiotherapy and molecularly targeted therapies. Response to first-line therapy is observed in 30–40% of patients, second line of treatment is even less effective.

Docetaxel is a well-known antimitotic drug that can be used in second-line treatment after failure of prior platinum based chemotherapy. It reversibly binds to microtubules (β-tubulins), stabilizes them and prevents their depolymerization. It also leads to phosphorylation of BCL-2, which is an apoptosis-blocking oncoprotein. Therefore, it works by interfering with cell division and inducing cell apoptosis. Docetaxel is effective in about 10% of patients.

The multidrug resistance (MDR) remains a substantial problem in development of resistance to chemotherapeutic treatment. The overexpression of ATP-binding cassette (ABC) transporters is responsible for cell removal of several classes of chemotherapeutics including taxanes, *vinca* alkaloids and nucleoside analogs, as well as physiologic substrates including leukotrienes and glutathione. The C subfamily of ABC proteins is alternatively known as the ABCC proteins or the multidrug resistance protein (MRP) subfamily. P-glycoprotein, a pivotal member of ABC transporters, is the product of multidrug resistance gene (*ABCB1/MDR1*). Polymorphisms (including single nucleotide polymorphisms, SNPs) of *MDR* genes are responsible for differences in expression and activity of several proteins involved in the removal of cytostatics form tumor cells.

The purpose of this study was to investigate potential clinical and molecular factors, including *MDR* genes polymorphisms, that could be used in qualification for second-line docetaxel therapy in patients with NSCLC after failure of first-line therapy.

## Material and Methods

The study group comprised of 58 Caucasian patients with locally advanced or metastatic NSCLC after failure of first-line chemotherapy. The staging of disease was determined according to the TNM classification (VII edition), and response to treatment was evaluated according to RECIST version 1.1. Performance status of patients was assessed in ECOG-WHO scale. All patients received first-line treatment with platinum-based chemotherapy. In all patients docetaxel at a dose of 75 mg/m^2^ was used as a second line treatment. Detailed characteristics of the patients are presented in Table 1.

**Table 1:** Characteristics of patients and influence of clinical factors on response to second-line docetaxel therapy in NSCLC patients

Factors	Cases	Response to treatment				Overall survival		
		SD, PR	PD	*p*	OR 95%CI	Median OS (months)	*p*	HR (95%CI)
Whole group	58	14 (24.1%)	44 (75.9%)			4.25		
Gender								
Male Female	49 (84.5%) 9 (15.5%)	11 (22.4%) 3 (33.3%)	38 (77.6%) 6 (66.7%)	0.7813	1.1633 0.7146–1.8911	4.5 3.5	0.5061	0.8234 (0.4642–1.4603)
Age (years)								
Median (± standard deviation) ≤ 65 > 65	61.7 (±7.7) 34 (58.6%) 24 (41.4%)	8 (23.5%) 6 (25%)	26 (76.5%) 18 (75%)	0.8551	1.0196 0.7577–1.372	4.5 6	0.6642	1.2048 (0.5194–2.7948)
Pathomorphological diagnosis								
Squamous cell carcinoma Adenocarcinoma Large cell carcinoma* NOS (*not-otherwise specified*) NSCLC*	30 (51.7%) 16 (27.6%) 6 (10.3%) 6 (10.3%)	10 (33.3%) 1 (6.3%)	20 (66.7%) 15 (93.7%)	0.0677	7.5 0.8633–65.1592	5 4.25	0.7716	1.1038 (0.5666–2.1504)
Stage of disease								
IIIB IV	19 (32.8%) 39 (67.2%)	7 (36.8%) 7 (17.9%)	12 (63.2%) 32 (82.1%)	0.2109	0.7697 0.5298–1.1183	6.5 3.5	0.1231	0.6322 (0.3529–1.1323)
Performance status (PS)								
PS > 1 PS ≤ 1	23 (39.7%) 35 (60.3%)	11 (47.8%) 3 (8.6%)	12 (52.2%) 32 (91.4%)	0.0019	0.5707 0.3809–0.8549	3 8	0.0001	3.4732 (1.8949–6.3663)
Previous surgical treatment ^b^								
Yes No	5 (8.6%) 53 (91.4%)	1 (20%) 13 (24.5%)	4 (80%) 40 (75.5%)	0.7486	1.06 0.6662–1.6865			
Previous radiotherapy								
Yes No	23 (39.7%) 35 (60.3%)	7 (30.4%) 7 (71.9%)	16 (69.6%) 28 (28.1%)	0.5520	0.8696 0.6333–1.194	5 3.5	0.5964	0.8561 (0.4817–1.5214)
Best response (first-line treatment)								
PD CR, PR, SD	17 (29.3%) 41 (70.7%)	2 (11.8%) 12 (29.3%)	15 (88.2%) 29 (70.7%)	0.2797	3.5753 0.9595–1.6218	2.5 6	0.0793	1.8728 (0.9291–3.7748)
Duration of best response (to first-line treatment, months)								
> 12 months ≤ 12 months	6 (10.3%) 52 (89.7%)	3 (50%) 11 (21.2%)	3 (50%) 41 (78.8%)	0.2893	0.6341 0.2814–1.429	8.75 3.5	0.1213	0.5457 (0.2536–1.1741)
Time from diagnosis to docetaxel therapy (months), Median (± standard deviation)								
> 12 ≤ 12	9 (± 7.6) 22 (37.9%) 36 (62.1%)	9 (40.9%) 5 (13.9%)	13 (59.1%) 31 (86.1%)	0.0437	0.6862 0.4732–0.9951	3 7.5	0.1193	1.5833 (0.8881–2.8225)
Weight loss (>5%) before docetaxel therapy								
Yes No	18 (31%) 40 (69%)	1 (5.6%) 13 (32.5%)	17 (94.4%) 27 (67.5%)	0.0592	1.3992 1.0979–1.7832	2.5 6	0.0015	3.5057 (1.6132–7.6182)
Anemia before docetaxel therapy								
No Yes	46 (79.3%) 12 (20.7%)	5 (41.7%) 9 (19.6%)	7 (58.3%) 37 (80.4%)	0.2245	0.7252 0.4403–1.1945	4.5 3.5	0.7062	0.8786 (0.4482–1.7222)
Toxicities of docetaxel therapy								
No Yes	38 (65.5%) 20 (34.5%)	5 (25%) 9 (23.7%)	15 (75%) 29 (76.3%)	0.8325	0.9828 0.7216–1.3384	4.5 3	0.0791	0.5532 (0.2857–1.0712)
Subsequent systemic treatment after docetaxel termination^a^								
No Yes	40 (69%) 18 (31%)					3 7	0.0059	2.2527 (1.2639–4.0151)

*not included in the statistical analysis

^a^including 13 patients treated with erlotinib without molecular selection

^b^not included in the survival analysis (subgroup of patients, who underwent surgical treatment was not large enough)

*TUBB3* gene expression was evaluated on RNA isolated from formalin fixed paraffin embedded tumor tissue. Isolation of RNA was carried out using the RNeasy FFPE Kit (Qiagen, Canada). SNPs of *ABCC2/MRP2* (rs12762549, g.99861014C > G) and *ABCB1/MDR1* (rs1045642, c.3435 T > C) genes were studied on DNA isolated from peripheral blood leucocytes, using the QIAmp Blood Mini Kit (Qiagen, Canada). Evaluation of the quality and quantity of extracted DNA/RNA was carried out using the BioPhotometer plus (Eppendorf, Germany).

*TUBB3* gene expression was assessed using Real-Time Quantitative Reverse Transcription PCR. Reverse transcription reaction was carried out using High Capacity cDNA Reverse Transcription Kit (Life Technologies, USA) with specific primers and reverse transcriptase. Expression of mRNA for *TUBB3* gene was analyzed on ABI PRISM 7500 equipment (Life Technologies, USA) using TaqMan Gene Expression Assay (Life Technologies, USA).

The evaluation of SNPs of *ABCC2/MRP2* and *ABCB1/MDR1* genes was performed using the PCR HRM (High Resolution Melting) technique. Reaction parameters for PCR and HRM were based on the manufacturer’s instructions from the KAPA FAST HRM PCR set (Kapa Biosystems, USA). PCR HRM was carried out on Eco Illumina (Illumina, USA) real-time PCR equipment.

Statistical analysis of data was performed using the MedCalc 10 (MedCalc Software, Belgium) and Statistica 10 (Statsoft, USA) software. Hardy-Weinberg equilibrium and the impact of demographic, clinical and genetic factors on the response to treatment and 6-month survival of the patients was estimated with the use of the Chi Square (χ^2^) test. Cox regression model with a stepwise selection of clinical and molecular factors with the minimum AIC was used to determine the model having the greatest impact on overall survival. Kaplan-Meier estimation method was used to compare the probability of overall survival in patients with different clinical, demographic and genetic characteristics. Large cell carcinoma and NOS (*not-otherwise specified*) NSCLC were excluded from statistical analysis due to its confusing nature and small number of cases. Also smoking status was excluded due to the fact, that. Most of patients (94.8%) were classified as heavy smokers. In all the performed test, results with *p* value of <0.05 were considered as statistically significant.

## Results

In the distribution of genotypes of the *ABCC2* and *MDR1* genes there were no deviations from the Hardy-Weinberg equilibrium. CC genotype of *ABCC2* gene was present in 21.1%, CG in 38.5% and GG in 40.4% of patients. In the case of the *MDR1* gene, genotypes CC, CT and TT were found in 15.4%, 44.2% and 40.4% of subjects. The distribution of genotypes of *ABCB1/MDR1* and *ABCC2/MRP2* genes did not depended on demographic and clinical factors.

The median value of ΔCt for *TUBB3* gene (relative to the reference gene *ACTB* – β-actin) was 5.9 cycles (± 5.41 cycles). Low expression of mRNA (below median value of ΔCt) for the *TUBB3* gene have been reported in 54.6%, while high expression in 45.4% of patients. Expression of mRNA for the *TUBB3* gene didn’t depended on demographic or clinical factors.

Among the studied patients, there were no complete remissions. Disease control was achieved in 14 patients (24.1%), 44 patients (75.9%) experienced early progression. Partial remission was observed in 4 patients (6.9%) In patients who reached disease control, median time to progression after docetaxel was 5 months (± 10.5 months).

Factors associated with reduced risk of early progression included: good PS (HR = 0.57, 95%CI: 0.38–0.85, *p* = 0.0019), long (>12 months) period between diagnosis of NSCLC and docetaxel treatment (HR = 0.69, 95%CI: 0.47–0.99, *p* = 0.0437). Disease control occurred significantly more often (HR = 0.71, 95%CI: 0.52–0.98, *p* = 0.0323) in patients with GG genotype of the *ABCC2/MRP2* gene than in carriers of other genotypes. For the remaining analyzed genes there was no significant difference in the incidence of progression or disease control depending on the presence of studied SNPs. Similarly, there was no significant difference in response to treatment depending on the level of expression of the *TUBB3* gene. Relationships between clinical and molecular factors and occurrence of early progression are presented in Table 2.

**Table 2:** Influence of genetic alterations on response to second-line docetaxel therapy in NSCLC patients

Factors	Cases	Response to treatment				Overall survival		
		SD, PR	PD	*p*	OR 95%CI	Median OS (months)	*p*	HR (95%CI)
*MDR1* (c.3435 T > C)								
CC CT TT	8 (15.4%) 23 (44.2%) 21 (40.4%)	2 (25%) 3 (13%) 4 (19%)	6 (75%) 20 (87%) 17 (81%)	0.7163	-	7 3.5 4.5	0.5541	-
*MDR1* (c.3435 T > C)								
CC CT or TT	8 (15.4%) 44 (84.6%)	2 (25%) 7 (15.9%)	6 (75%) 37 (84.1%)	0.5357	1.7619 0.2934–10.5811	7 3.5	0.6007	0.7990 0.3448–1.8516
*MDR1* (c.3435 T > C)								
TT CC or CT	21 (40.4%) 31 (59.6%)	4 (19.1%) 5 (16.1%)	17 (80.9%) 26 (83.9%)	0.7851	1.2235 0.287–5.216	3.75 3.5	0.6050	0.8510 0.4616–1.5686
*ABCC2* (g.99861014C > G)								
CC CG GG	11 (21.1%) 20 (38.5%) 21 (40.4%)	1 (9.1%) 1 (5%) 7 (33.3%)	10 (90.9%) 19 (95%) 14 (66.7%)	0.0407	-	4.5 3 4.5	0.6660	-
*ABCC2* (g.99861014C > G)								
CC CG lub GG	11 (21.1%) 41 (78.9%)	1 (9.1%) 8 (19.5%)	10 (90.9%) 33 (80.5%)	0.4293	0.4125 0.0459–3.7079	3.5 4.5	0.6211	1.2070 0.5724–2.5450
*ABCC2* (g.99861014C > G)								
GG CC or CG	21 (40.4%) 31 (59.6%)	7 (33.3%) 2 (6.5%)	14 (66.7%) 29 (93.5%)	0.0221	7.2500 1.3297–39.5283	4.5 3.5	0.3361	0.7426 0.4049–1.3619
*TUBB3* mRNA expression (ΔCT)								
Low High	22 (42.3%) 30 (57.7%)	6 (27.3%) 3 (10%)	16 (72.7%) 27 (90%)	0.1162	3.3750 0.7399–15.3946	8 4	0.7539	0.8486 0.3041–2.3682

The median overall survival was 4.25 months (± 9.6 months). In 24 patients (41.4%) survival was longer than 6 months. Factors such as: good PS (HR = 0.26, 95%CI: 0.12–0.58, *p* < 0.0001), disease control after docetaxel (HR = 0.091, 95%CI: 0.014–0.63, *p* < 0.0001), long period from diagnosis to docetaxel (HR = 0.35, 95%CI: 0.17–0.71, *p* = 0.0004), lack of significant weight loss prior to treatment (HR = 0.48, 95%CI: 0.32–0.73, *p* = 0.0021) and the use of third-line treatment (HR = 0.48, 95%CI: 0.24–0.94, *p* = 0.0195) were associated with prolongation of survival beyond 6 months. Other clinical factors did not have a significant effect on the 6 months survival. We found no significant impact of individual SNPs or *TUBB3* gene expression on the survival longer than 6 months.

Overall survival probability estimated by the Kaplan-Meier method was lower in patients with significant weight loss (HR = 3.51, 95CI%: 1.61–7.62; *p* = 0.0015), poor PS (HR = 3.47, 95CI%: 1.89–6.37; *p* < 0.0001), lack of disease control after docetaxel (HR = 2.89, 95CI%: 1.60–5.21; *p* = 0.0004), and unable to receive third-line treatment (HR = 2.25, 95CI%: 1.26–4.01; *p* = 0.0059). There was no significant impact of individual SNPs as well as *TUBB3* gene expression on overall survival probability.

**Fig. 1:**
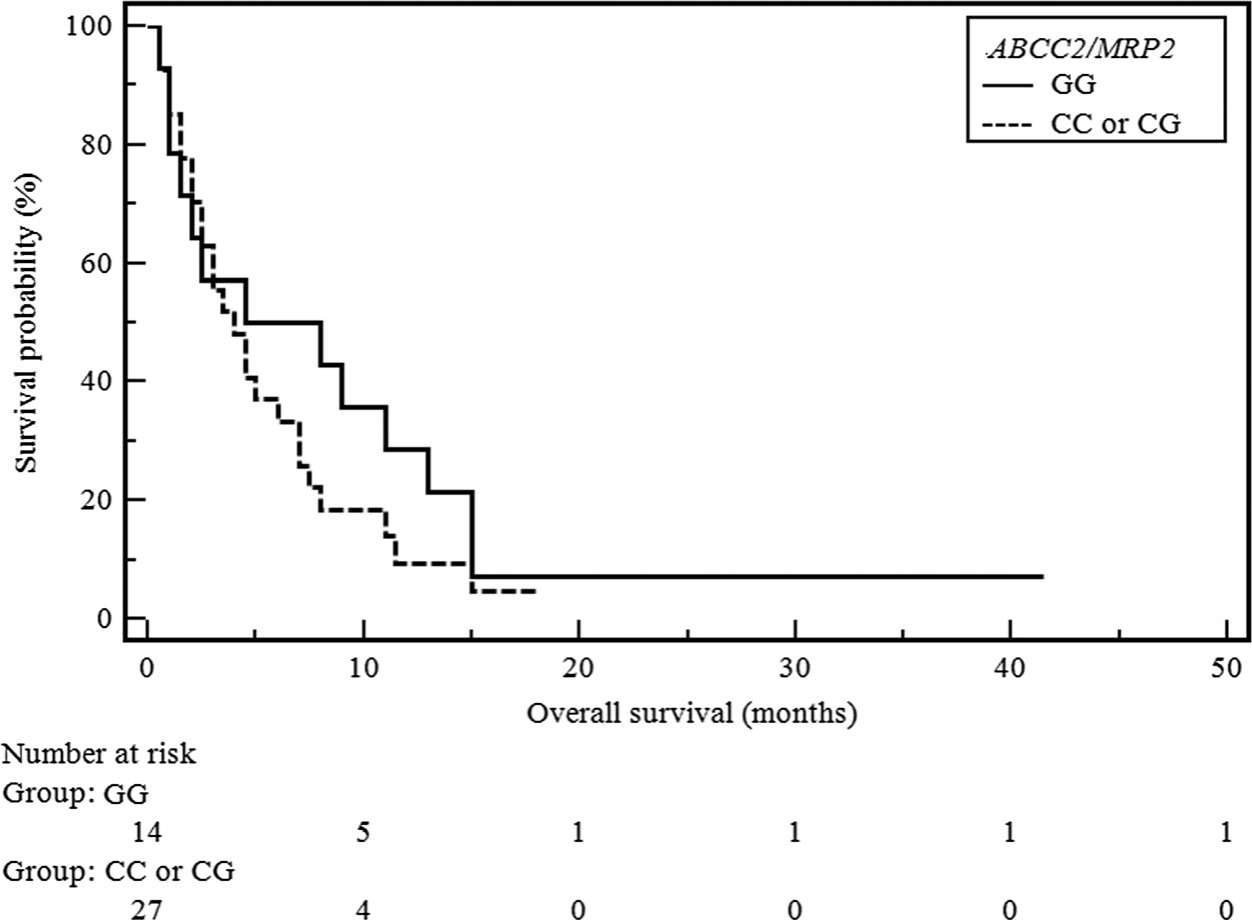
Survival curves of NSCLC patients with different *ABCC2/MRP1* genotypes

**Fig. 2:**
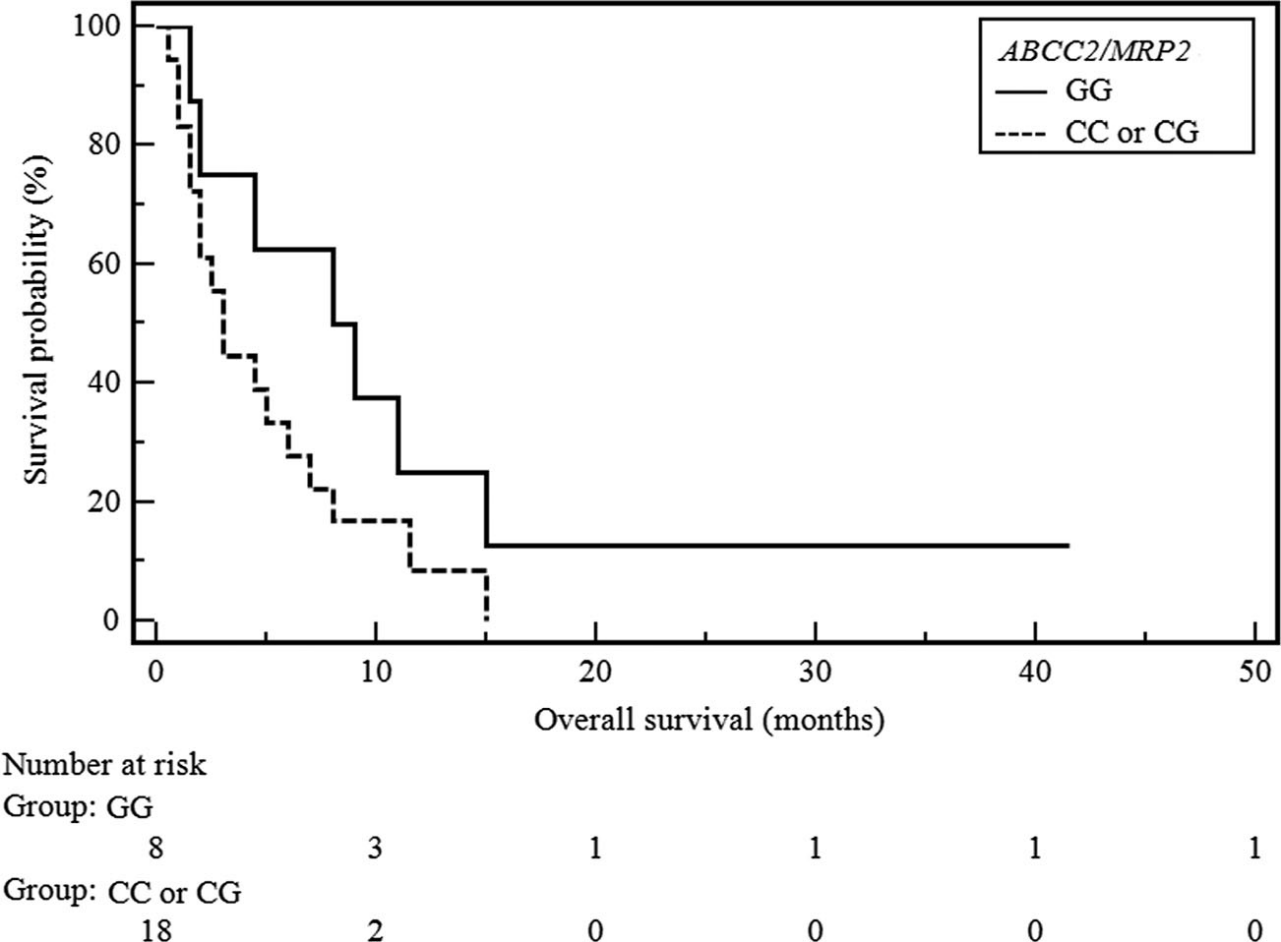
Survival curves of SCC patients with different *ABCC2/MRP1* genotypes

Although it seems that the applied treatment has a higher efficacy in the SCC subgroup of patients, we have failed to demonstrate a statistically significant effect on overall survival (Table 3). Kaplan-Meier survival curves of NSCLC and SCC patients with different *ABCC2/MRP1* genotypes were shown in Figs. 1 and 2.

**Table 3:** Influence of genetic alterations on response to second-line docetaxel therapy in subgroup of SCC patients

Factors	Cases	Response to treatment				Overall survival		
		SD, PR	PD	*p*	OR 95%CI	Median OS (months)	*p*	HR (95%CI)
*MDR1* (c.3435 T > C)								
CC CT TT	6 (20%) 14 (46.7%) 10 (33.3%)	2 (33.3%) 2 (14.3%) 3 (30%)	4 (66.7%) 12 (85.7%) 7 (70%)	0.5421	-	8 2 4.5	0.8798	-
*MDR1* (c.3435 T > C)								
CC CT or TT	6 (20%) 24 (80%)	2 (33.3%) 5 (20.8%)	4 (66.7%) 19 (%)	0.5215	1.9 0.2669–13.5235	8 4.5	0.6320	0.7224 0.1909–2.7335
*MDR1* (c.3435 T > C)								
TT CC or CT	10 (46.7%) 20 (53.3%)	3 (30%) 4 (20%)	7 (70%) 16 (80%)	0.5439	1.7143 0.3007–9.7731	4.5 4.5	0.9511	0.9736 0.4138–2.2905
*ABCC2* (g.99861014C > G)								
CC CG GG	7 (23.3%) 14 (46.7%) 9 (30%)	1 (14.3%) 1 (7.1%) 5 (55.6%)	6 (85.7%) 13 (92.9%) 4 (44.4%)	0.0224	-	3 3 8.5	0.3521	-
*ABCC2* (g.99861014C > G)								
CC CG lub GG	7 (23.3%) 23 (76.7%)	1 (14.3%) 6 (26.1%)	6 (85.7%) 17 (73.9%)	0.5248	0.4722 0.0468–4.7697	3 4.5	0.6527	1.2863 0.4296–3.8518
*ABCC2* (g.99861014C > G)								
GG CC or CG	9 (30%) 21 (70%)	5 (55.6%) 2 (9.5%)	4 (44.4%) 19 (90.5%)	0.0135	11.875 1.6684–84.5221	8.5 3	0.1227	0.5051 0.2122–1.2024
*TUBB3* mRNA expression (ΔCT)								
Low High	14 (46.7%) 16 (53.3%)	5 (35.7%) 2 (12.5%)	9 (64.3%) 14 (87.5%)	0.1483	3.8889 0.6168–24.5182	8 4	0.6164	0.7114 0.1878–2.6956

According to the Cox model (general model fit: *p* = 0.0024) demographic, clinical and genetic factors that characterized the highest risk of survival shortening were: lack of third-line treatment (HR = 23.89, 95%CI: 3.50–163.02, *p* = 0.0013), lack of best response to first-line therapy (HR = 11.34, 95%CI: 2.04–63.11, *p* = 0.0058), poor PS (HR = 7.06, 95%CI: 1.59–31.32, *p* = 0.0105), and CG or GG genotypes of *ABCC2/MRP2* gene (HR = 5.21, 95%CI: 1.08–25.05, *p* = 0.0404).

## Discussion

TAX 317 [1] and TAX 320 [2] studies demonstrated effectiveness of docetaxel after failure of first-line chemotherapy. In the TAX 317 study, both the median survival (7.5 months) as well as the 12 month survival rate (37%) were significantly higher in patients treated with docetaxel compared to patients receiving best supportive care (4.6 months and 11%, retrospectively). In the TAX 320 study, response occurred in 7.5–11.9% of patients depending on the dose of docetaxel, and in 1% of patients in the control group receiving vinorelbine or ifosfamide. 12 month survival rate was 32% in patients treated with docetaxel and 19% in the control group. Second line treatment can be used in patients with PS scored at 0–1, and in exceptional cases with PS = 2. Patients with PS > 2 should not be treated [3]. The results obtained in our study confirm that PS is critical, affecting all of the analyzed parameters of treatment effectiveness: the risk of early progression, overall survival, and risk of death. The median survival of patients in good PS (PS ≤ 1) was 8 months, while patients in worse condition (PS > 1) only survived 3 months.

One of the few studies which address the problem of determining predictors of benefit from second-line treatment in NSCLC is a phase III study by Hanna et al. [4] comparing the efficacy of docetaxel and pemetrexed. Analysis showed significantly longer survival among women, patients with stage IIIB, in good PS (PS ≤ 1) and with objective response to first-line treatment. Unfortunately, the study involved patients who qualified under strict criteria – 89% of them were in good or very good PS (PS ≤ 1). Median survival for patients with PS = 0 was 12.7 months, compared to 8.3 months for patients with PS = 1 and 2.6 months for PS = 2. Median survival was 9.4 months for women, and 7.2 months for men. Similar differences were observed in patients differentiated by stage of disease (IIIB vs. IV). Patients with objective response to first-line treatment achieved longer survival than patients with stable disease (median 15.8 vs 10.5 months), and patients from both above groups fared better than those who progressed (median survival of 4.6 months).

In the univariate analysis, Weiss et al. [5] found a longer median survival in patients with non-squamous NSCLC and with first-line response >3 months. In authors study PS of 60.3% of patients was rated as 2, which resulted in shorter median survival. Multivariate analysis showed that in addition to the factors mentioned in the analysis by Weiss et al., survival was also affected by substantial weight loss (>5%), male sex, lack of surgical treatment prior to chemotherapy and third-line treatment after docetaxel.

Tibaldi et al. [6] treated older patients (70–83 years, median 74 years) with reduced dose (37.5 mg/m^2^) of docetaxel. Despite the reduction 21% of patients reached partial remission, and another 36% stable disease. Those results were better than in both TAX studies. This probably isn’t associated with greater effectiveness of docetaxel in this age group, but with slower course of the disease in the elderly.

In a study evaluating the effect of prior first-line treatment on effectiveness of second-line docetaxel in patients with NSCLC, Macedo-Pérez et al. [7] showed that long (>6 months) response to first-line treatment is associated with significant increase in time to progression (TTP) and overall survival.

Our results confirmed crucial predictive role of such factors as: poor PS, significant weight loss and short time from diagnosis to docetaxel treatment. However, we have found no effect of gender, age, stage of disease or pathological diagnosis on overall survival of NSCLC patients treated with docetaxel. Interpretation of impact of the third-line treatment after docetaxel is difficult. We found that third-line therapy can significantly increase the probability of survival longer than 6 months. However, such treatment is used in patients with good PS, which is the most important predictive factor.

In the absence of independent predictors that could help qualify for second-line docetaxel treatment, currently the biggest hope rests on molecular research. It has been shown that patients with high expression of class III β-tubulin benefit less from paclitaxel treatment [8]. In meta-analysis of 10 clinical trials in which NSCLC was treated with paclitaxel and/or vinorelbine, Zhang et al. [9] demonstrated a correlation between low expression of mRNA for the *TUBB3* gene and favorable outcomes – in the group of patients with low expression percentage of objective response was higher, and median survival longer. Kaira et al. [10] evaluated effects of class III β-tubulin expression on progression-free survival and overall survival in patients with NSCLC treated with taxanes. Researchers demonstrated that in patients treated with docetaxel high *TUBB3* expression is predictive for short time to progression and concluded that it is associated with resistance to taxanes. In our study no significant difference in response to therapy, probability of survival longer than 6 months, or overall survival was found depending on the level of mRNA expression of the *TUBB3* gene.

In normal lung, ABCB1 is expressed on the surface of bronchi and at the plasma membrane of alveolar macrophages. In lung cancer, ABCB1 expression is initially low, but this may change after exposure to chemotherapy as part of acquired drug resistance. ABCB1 confers resistance to cytotoxic drugs, including etoposide and cisplatin. ABCC2 is expressed at low levels in normal lung tissue and at high levels in lung cancer. It has higher expression in poorly differentiated compared to well-differentiated tumors. This suggests that these tumors may quickly develop resistance to anti-cancer agents. In tumor cell lines, ABCC2 mRNA overexpression was associated with resistance to etoposide, vincristine, cisplatin, doxorubicin and epirubicin.

Haenish S et al. [11] showed that *ABCB1MDR1* and *ABCC2/MRP2* SNPs modulate the expression of multidrug resistance protein in the unaffected renal cortex of renal cell carcinoma patients. In Meyer et al. [12] study, high MRP2 expression was correlated with *ABCC2* gene variants (especially rs717620) in liver tissue. Such correlation was not found between *ABCB1/MDR1* SNPs and MDR1 expression. Allelic variants of the *ABCC2/MRP2* gene have also been associated with toxicity induced by chemotherapy agents and lung cancer survival. Few reports suggest that polymorphisms in *ABCB1/MDR1* significantly influence the therapeutic response in lung cancer, although these findings are not always concordant. Therefore, *MDR* genes polymorphisms possibly contribute to inter-individual differences in drug and xenobiotics elimination and chemotherapy efficacy. However, the role of *MDR* genes SNPs in docetaxel susceptibility or resistance needs further investigation in non-small cell lung cancer patients.

Previous studies related to polymorphisms of *MDR* genes and chemotherapy effectiveness were conducted mainly in the populations of Asian origin. Isla et al. [13] studied the effects of SNPs of *ERCC1*, *XPD*, *RRM1*, *ABCB1/MDR1* genes, and mRNA expression for *ERCC1* on overall survival in patients with advanced NSCLC treated with docetaxel and cisplatin. Authors ascertained that evaluation of *ERCC1* gene SNPs may be an important factor for effectiveness of platinum-based chemotherapy. However, analysis of the *MDR1* gene polymorphisms showed no statistically significant results.

In their 2008 paper, Kiyotani et al. [14] studied the effects of SNPs in seven genes potentially associated with transport and metabolism of docetaxel (*CYP3A4*, *CYP3A5*, *ABCB1*/*MDR1*, *ABCC2/MRP2*, *SLCO1B3*, *NR1I2* and *NR1I3*) in Japanese patients with NSCLC. Polymorphisms in *ABCC2* (rs12762549) and *SLCO1B3* (rs11045585) genes were significantly associated with the risk of docetaxel induced leucopenia. In combined evaluation of both polymorphisms, patients with unfavorable genotype proved to have significantly higher risk of neutropenia compared to patients with favorable genotype. Assessment system based on the genotyping of patients allowed to correctly classify 69.2% of patients with severe neutropenia and 75.7% of patients without this adverse effect to the appropriate categories, confirming that the SNPs in *ABCC2* and *SLCO1B3* genes may be a predictive factor for docetaxel induced neutropenia.

In a paper describing Caucasian patients, Campa et al. [15] characterized the genetic variability of genes *ABCB1*/*MDR1*, *ABCC2* and *ABCG2* in lung cancer patients treated with chemotherapy. Of the studied polymorphisms, SNP rs717620 of the *ABCC2* gene has been linked to differences in treatment response, progression-free survival and overall survival of patients with small cell lung cancer (SCLC), but this effect was not observed in NSCLC. Lewis et al. [16] in their study which included 64 American NSCLC patients receiving docetaxel showed that SNP rs12762549 of the *ABCC2* gene is associated with reduced clearance of the docetaxel. In our study, in the model including demographic, clinical and genetic factors, SNP rs12762549 of the *ABCC2* gene (C/G and G/G genotypes) was one of the factors that had a significant impact on the risk of shortened overall survival.

## Conclusions

Based on the results of our study it can be concluded that second-line docetaxel monotherapy in the treatment of NSCLC has limited efficacy, and qualification of patients for this type of treatment requires a careful analysis of available prognostic and predictive factors. Factors assessed in this study mainly have prognostic and not predictive value, which is consistent with the results of other researchers. Molecular studies, especially of the genes whose protein products are responsible for the clearance of cytostatics from tumor cells, may be useful in the selection of patients for docetaxel monotherapy. Finding reliable molecular predictors for this type of therapy however requires randomized, prospective clinical trials.
